# Gastrectasis B—Diagnosis

**Published:** 1891-08-22

**Authors:** 


### The Practitioner's Mirror and Retrospect.

[*The Editor will be glad to receive offers of co-operation and contributions from members of the profession. All letters should be addressed to* The Editor, The Lodge, Porchester Square, London, W.]

### MEDICINE.

### GASTRECTASIS (*continued*).

B.—Diagnosis.

Professor Dujardin-Beaumetz, in a recent lecture at the Hôpital Cochin, dwelt on the relationship between gastric neurasthenia and gastrectasis, a subject to which we shall refer fully in a future article. But as regards the diagnosis of dilatation of the stomach, he considers the most important sign is the existence of the *bruit de clapotement*. He states that it is not always easy to perceive this *bruit de clapotement*, even in the case of dilatations that are considerable. For this there are two special reasons: (1) An empty stomach; (2) chiefly the contraction of the muscles of the abdomen, and in particular of the recti muscles. It is easy to remedy the first cause by making the patient drink a glass of cold water while fasting, but it is more difficult to overcome the second cause. One may generally succeed, however, by making the patient take a deep breath, or by pressing suddenly with one or two fingers over the abdominal wall, or by seizing with both hands the two sides of the abdomen, imparting to the whole belly sudden movements, just as we should do in order to obtain hippocratic succussion in hydro-pneumo-thorax, when the characteristic plashing sound will be detected. If we listen for the sound before the water is taken, the difference in the two conditions may be very obvious. The professor states that in order to diagnose dilatation of the stomach, this bruit must be perceived below the oblique line which passes from the borders of the false ribs on the left side to the umbilicus. Any *bruit de clapotement* heard above this line must be considered as physiological. He further lays stress on the importance of examining the iliac fossæ. Often one will find there a bruit of gaseous collision, which, by its *timbre*, may be differentiated from the *bruit de clapotement*. This bruit is evidence of dilatation of the large intestine. Following his directions, we have found these procedures most useful in several cases under our care. Dr. Wolff,* in his paper, to which we have already referred, and which we have made to some extent the basis of our article, states that he has used auscultation with great advantage in the diagnosis of gastrectasis, if combined with a tube and inflation of the stomach containing fluids. This is especially of service in determining the lower segment of the dilated stomach and extent of the distension. To this end he employs an ordinary small stomach tube, having previously ascertained that the stomach was empty, or if not so, having washed it out, the patient is directed to take a glass of water. If, now, the stomach is inflated while the ear is applied to the epigastrium, the point of greatest intensity of the bubbling sound of the air escaping through the small quantity of water contained in the stomach will accurately determine the lowest part of the stomach. In patients with these abdominal parietes this will become readily apparent to palpation. The swallowing sounds, or "schluck geräusche," so important at times in the study of cardiac constriction, are of little, if any, importance in the study of gastric dilatation.

We think that these diagnostic procedures, the importance of which Dujardin-Beaumetz and Wolff have emphasised, will be of great assistance in determining accurately the presence and extent of dilatation of the stomach, for the physical signs upon which till recently we had to rely, have been so indefinite as to prove almost useless. Percussion of course does render some assistance, but as the large intestine is very frequently considerably distended, we can never feel absolute confidence in the so called typical percussion note of the stomach, more especially when we desire to accurately map out the limits of the distended viscus.

We have recently discussed the methods by which the presence of free hydrochloric acid in the stomach may be determined, and its percentage estimated, and, therefore, need not enter fully this point; but it must be noted that although the presence or absence of this acid is of small value in diagnosing the existence of cancer, it is of great importance in determining the degree of degeneration of the gastric mucosa in the disease under consideration, and in arriving at a reliable prognosis. Wolff states that most favourable are the atonic cases of recent distension, owing to continued errors of diet and excessive eating and drinking. In these cases, which, for convenience, he terms paretic ectasis of the stomach, good results will probably always follow proper treatment. As much cannot probably be said of cases where the distension is complete, and the muscular fibres are in a state of paralysis. Under such circumstances, which are characterised by the continued absence of hydrochloric acid from the stomach, much can be done to relieve the distress consequent thereupon, together with the dyspeptic symptoms, and a lack of the proper elaboration of chymus in the intestines, and the mal-assimilation depending upon it. Little is to be hoped for reducing the viscus to its normal dimensions, or establishing in such stomachs a proper chemical digestive act. Such patients, he finds, may be kept for years in a comfortable condition by observing a proper regimen or avoiding errors in diet.

* *Therap. Gaz.*, July, 1891.

